# History of U.S. Iodine Fortification and Supplementation

**DOI:** 10.3390/nu4111740

**Published:** 2012-11-13

**Authors:** Angela M. Leung, Lewis E. Braverman, Elizabeth N. Pearce

**Affiliations:** Section of Endocrinology, Diabetes, and Nutrition, Boston Medical Center, 88 East Newton Street, Evans 201, Boston, MA 02118, USA; Email: lewis.braverman@bmc.org (L.E.B.); elizabeth.pearce@bmc.org (E.N.P.)

**Keywords:** history, iodine, supplementation

## Abstract

Iodine is a micronutrient required for thyroid hormone production. This review highlights the history of the discovery of iodine and its uses, discusses the sources of iodine nutrition, and summarizes the current recommendations for iodine intake with a focus on women of childbearing age.

## 1. Importance of Adequate Iodine Nutrition

Adequate levels of iodine, a trace element variably distributed on the earth and found mostly in the soil and water of coastal areas, are required for the synthesis of the thyroid hormones, thyroxine (T4) and triiodothyronine (T3), which play key roles in the metabolic processes of vertebrate life. The major concerns regarding the global burden of iodine deficiency are related to goiter, neurocognitive impairments, and in severe deficiency, hypothyroidism resulting in cretinism.

## 2. Discovery and Early Investigations of Iodine

Early Chinese medical writings in approximately 3600 B.C. were the first to record the decreases in goiter size upon ingestion of seaweed and burnt sea sponge [1]. Although iodine was yet to be discovered, these remedies remained effective and their use continued globally, as was documented in writings by Hippocrates, Galen, Roger, and Arnold of Villanova in later centuries [1].

The discovery of iodine was made incidentally during the early part of the 19th century. In 1811, while extracting sodium salts necessary for the manufacture of gunpowder, Bernard Courtois, a French chemist, observed an unusual purple vapor arising from seaweed ash treated with sulphuric acid [1]. Studies of this previously unrecognized substance were continued by Joseph Louis Gay-Lussac, Andre Anpere, Sir Humphry Davy, and others. In 1813, the first paper presenting the new element, iodine (termed after the Greek word, *ioeides*, translated as violet-colored), by Gay-Lussac was read [1]. Shortly following this, J.F. Coindet, a physician in Switzerland, published his observations that administration of iodine (as grains in distilled alcohol) was able to decrease the size of his patients’ goiters [2]. In 1852, Adolphe Chatin, a French chemist, was the first to publish the hypothesis of population iodine deficiency associated with endemic goiter [3]. This was confirmed by Eugen Baumann, who, in 1896, reported the discovery of iodine within the thyroid gland [4]. 

## 3. Sources of Iodine Intake and Exposure

Iodine (atomic weight 126.9 g/atom) is present in the upper crust of the earth as a trace element. The effects of glaciation, flooding, and leaching into soil during the Ice Age have led to the variable geographic distribution of iodine. As a result of these natural forces, iodine accumulation is found mostly in coastal areas, and the most common sources of dietary iodine are seaweed and other seafood. 

The fortification of salt with iodine is an effective, inexpensive, and stable route of ensuring adequate iodine intake. The stability of iodine in salt may depend on various environmental and storage conditions [5]. In some regions of the world where salt iodization is impractical, iodization of other common foods (bread and others) is targeted, or individuals receive administered oral or intramuscular iodized oil supplements. Other sources of intermittent iodine exposure may include the use of iodine-rich medications (such as amiodarone and iodine-enriched cough medications), topical antiseptics, iodine-containing multivitamins and supplements, radiographic contrast agents, and water purification tablets.

## 4. Dietary Iodine

In the U.S., iodine is present in dairy foods (due to the iodophor cleansers of milk cans and teats) and occasionally in bread dough (due to the use of iodate as bread conditioners). Iodine is only one of several teat dip formulations available in the industry [6] and represents an “accidental” but important source of iodine nutrition. Seafood is another excellent source of dietary iodine. The Total Diet Study by the U.S. Food and Drug Administration (FDA) in 2003–2004 reported that the important sources of dietary iodine were dairy and grain products [7], as was confirmed by a recent survey of these foods in the Boston area [8]. The iodine content of plant foods depends on the iodine levels in soil and in groundwater used in irrigation, in crop fertilizers, and in livestock feed. Iodine concentrations of plants grown in soils of iodine-deficient regions may be as low as 10 μg/kg of dry weight, in contrast to that of plants grown in iodine-rich areas, which may be as high as 1000 μg/kg dry weight [9]. Most foods contain 3–75 μg of iodine per serving [10].

## 5. Iodine Fortification and Supplementation in the U.S.

The results of early studies were the basis for advocating iodine supplementation to decrease goiter. In the 1830s, the French nutritional chemist, Jean Baptiste Boussingault, observed that the prevalence of goiter was increased in areas where naturally-occurring iodized salt was infrequently consumed and recommended the distribution of naturally iodized salt for public consumption [2]. However, although there were attempts to implement iodine prophylaxis in the U.S., Switzerland, France, and other areas, this was not widely adopted until many decades later, due to expense and the occurrence of iodine-induced hyperthyroidism (Jöd-Basedow phenomenon), especially in individuals with an underlying nodular goiter living in areas of iodine deficiency. Iodine supplementation, primarily through the fortification of table salt, did not begin until the early 1920s and occurred initially in Switzerland and the U.S.

Prior to the 1920s, endemic iodine deficiency was prevalent in the Great Lakes, Appalachians, and Northwestern regions of the U.S., a geographic area known as the “goiter belt”, where 26%–70% of children had clinically apparent goiter [11]. During the draft for World War I, a Michigan physician, Simon Levin, observed that 30.3% of 583 registrants had thyromegaly (including both toxic and nontoxic goiters), many of which were large enough to disqualify them from the military, in accordance with U.S. Selective Service regulations [12]. Subsequent surveillance studies in the following year by Levin and R.M. Olin, Commissioner of the Michigan State Department of Public Health, demonstrated that the prevalence of goiter reached as high as 64.4% in some parts of Michigan [12]. 

David Marine, a U.S. physician in Ohio, and his colleagues initiated an iodine prophylaxis program in over 2100 schoolgirls in 1917. Over the next few years, he and colleagues published a series of papers reporting a significantly decreased frequency of goiter in children treated with iodine (0.2%), compared to children who did not receive iodine supplementation (>25%) [13]. In 1922, David Cowie, chairman of the Pediatrics Department at the University of Michigan, proposed at a Michigan State Medical Society thyroid symposium that the U.S. adopt salt iodization to eliminate simple goiter [12]. His work with the Society over the next few years, through the development of the Iodized Salt Committee, was instrumental in the history of the U.S. iodine supplementation effort [12].

## 6. Iodized Salt

In the U.S., iodized salt first became available on grocery shelves in Michigan on 1 May 1924, spurred largely by the series of reports by Cowie, Marine, and others in the preceding few years [14]. The region had largely been severely iodine deficient, and Hartsock in 1926 described an outbreak of thyrotoxicosis in adults who took iodized salt living in the Great Lakes region of the goiter belt [15]. 

Salt was initially fortified with iodine at 100 mg/kg, resulting in an estimated average intake of 500 µg iodine daily [13]. However, many individuals in other states continued to resist efforts to make iodized salt freely available for the next several decades. Although a bill by the U.S. Endemic Goiter Committee in 1948 proposing the mandatory introduction of iodized salt in all states was defeated [13], the proportion of U.S. households which use only iodized salt has remained stable at 70%–76% since the 1950s [16]. Although the International Council for the Control of Iodine Deficiency Disorders (ICCIDD) Global Network estimates that the proportion of U.S. households with access to iodized salt now exceeds 90% [17], data regarding actual usage is limited and the contribution of iodized salt to the overall iodine sufficiency of the U.S. population is uncertain.

Salt iodization is a useful approach toward decreasing iodine deficiency in populations. It is a universal foodstuff, intake is seasonally consistent, costs are relatively small, and it is easily distributed [18]. Approximately 120 countries, including Canada and some parts of Mexico, have adopted mandatory iodization of all food-grade salt [5], although the extent of implementation efforts in individual countries is unknown. In contrast, fortification of salt with iodine in the U.S. is voluntary, and the FDA does not mandate the listing of iodine content on food packaging. Furthermore, it is assumed that the majority of salt consumption in the U.S. comes from processed foods, in which primarily non-iodized salt is used during production [19]. Although iodized salt in the U.S. is fortified at 45 mg iodide/kg, 47 of 88 table salt brands recently sampled contained less than the FDA’s recommended range of 46–76 mg iodide/kg [5].

## 7. Iodine in Supplements and Multivitamins

Iodine is required for normal brain myelination in utero and during the early post-partum period. As such, the developing fetus and infant are particularly vulnerable to the effects of inadequate iodine nutrition. The World Health Organization (WHO) recommends 250 μg of total iodine intake daily during pregnancy and lactation [20], and the U.S. Institute of Medicine recommends 220 μg daily during pregnancy and 290 μg during lactation, higher than the 150 μg required by non-pregnant adults [10].

Prenatal and other multivitamins marketed in the U.S. are not required to contain iodine. Data from the U.S. National Health and Nutrition Examination Survey (2001–2006) demonstrated that only 20.3% of pregnant women routinely take an iodine-containing supplement [21]. A recent survey of 223 prenatal multivitamins available in the U.S. reported that only 51% of the brands listed any iodine content (as either potassium iodide or kelp), and measured values may be discrepant from their labeled amounts, especially in those containing kelp [22]. In the U.S., the Dietary Supplement Health and Education Act (DSHEA) and the Dietary Supplement and Nonprescription Drug Consumer Protection Act provides the FDA with only enforcement authority to remove products that pose an immediate safety concern or an unacceptable risk of illness of injury; supplements are not monitored and regulated to the same rigorous standards as FDA-approved medications [23].

## 8. Current Status of and Recommendations for Iodine Nutrition

Levels of urinary iodine cannot be used to determine iodine status in an individual, given the day-to-day variation in dietary iodine intake. Median urinary iodine levels are used instead and reflect dietary iodine sufficiency across populations [5]. In the U.S., data from large population studies have shown that median urinary iodine levels decreased by approximately 50% between the early 1970s and the early 1990s, although the population overall remained iodine sufficient [24]. Subsequent studies have shown that this decrease has stabilized [25,26,27], although some subsets of the population, in particular pregnant women and women of childbearing age, may be at risk for mild to moderate iodine deficiency.

Iodine deficiency remains one of the most important public health issues globally, and an estimated 2.2 billion people live in iodine-deficient areas [28]. In 1990, the United Nations World Summit for Children set forth the goal of eliminating iodine deficiency worldwide [29], and considerable progress has since been achieved. This has largely been led by programs of universal salt iodization (USI) in various countries, in line with the recommendations by the World Health Organization (WHO) and the ICCIDD Global Network [17,20]. Other groups which have been instrumental in advocating for improved iodine nutrition have been Kiwanis International and the U.S. Center for Disease Control [16].

A public health approach has been used to help ensure adequate iodine intake in U.S. pregnant and lactation women. Guidelines by the American Thyroid Association and the Endocrine Society urge that prenatal vitamins for U.S. and Canadian women contain 150 μg potassium iodide (containing 114 μg iodine) daily during pregnancy and lactation [30,31], a recommendation that has been recently endorsed by the Neurobehavioral Teratology Society [32]. The U.S. National Academy of Sciences also advocates that iodine be included in all prenatal multivitamins [33].

## 9. Conclusions

The U.S. was historically iodine deficient prior to the early 1920s, particularly in the goiter belt region of the Great Lakes, Appalachians, and the northwestern area of the country, due to the effects of natural atmospheric processes. Following the successful implementation of salt iodization program in Switzerland, the introduction of iodized table salt in the U.S. during the 1920s significantly improved its iodine nutritional status. However, although recent national studies demonstrate that the general population is overall iodine sufficient, salt iodization in the U.S. is not universal, and certain subsets of the population, including pregnant and lactating women and their offspring, may be at risk for mild to moderate iodine deficiency. As such, a public health approach by the American Thyroid Association and the Endocrine Society advocate U.S. women to take a supplement containing 150 µg iodine/day beginning preconception.
