# Erratum: History of U.S. Iodine Fortification and Supplementation; *Nutrients* 2012, *4*, 1740–1746

**DOI:** 10.3390/nu9090976

**Published:** 2017-09-05

**Authors:** Angela M. Leung, Lewis E. Braverman, Elizabeth N. Pearce

**Affiliations:** 1 Division of Endocrinology, Diabetes, and Metabolism, UCLA David Geffen School of Medicine, 11301 Wilshire Blvd (111D), Los Angeles, CA 90073, USA; 2 Division of Endocrinology, Diabetes, and Metabolism, VA Greater Los Angeles Healthcare System, 11301 Wilshire Blvd (111D); Los Angeles, CA 90073, USA; 3 Section of Endocrinology, Diabetes, and Nutrition, Boston Medical Center, 88 East Newton Street, Evans 201, Boston, MA 02118, USA; lewis.braverman@bmc.org (L.E.B.); elizabeth.pearce@bmc.org (E.N.P.)

The authors requested the following corrections to their paper [1].

In the text of Section 3, “126.9 g/atom” was changed to “126.9 atomic mass unit (amu)”.

The authors apologize for any inconvenience caused to the readers by the change, stating it does not affect the scientific results. The original manuscript will be modified accordingly.
